# Cleaning protocols in forensic genetic laboratories

**DOI:** 10.1007/s00414-024-03232-0

**Published:** 2024-04-23

**Authors:** Marie-Louise Kampmann, Jacob Tfelt-Hansen, Claus Børsting

**Affiliations:** 1 Section of Forensic Genetics, Department of Forensic Medicine, Faculty of Health and Medical Sciences, University of Copenhagen, Copenhagen, Denmark; 2 The Department of Cardiology, The Heart Centre, Copenhagen University Hospital, Copenhagen, Denmark

**Keywords:** Laboratory cleaning, Contamination, DNA, PCR, qPCR, Forensic genetics

## Abstract

It is pivotal to avoid cross-sample contamination in forensic genetic laboratories and optimal cleaning protocols for the removal of DNA are essential. A survey was performed, and ten forensic genetic laboratories shared their cleaning protocols in pre-PCR and post-PCR laboratories. The cleaning frequencies on different surface areas were somewhat similar, whereas none of the laboratories used the same cleaning reagents. Therefore, the efficiencies of the cleaning protocol utilised were tested and compared. The results showed that freshly made household bleach and Virkon® removed all amplifiable DNA from the surfaces, whereas DNA AWAY™ and the disinfection reagents ethanol, isopropanol, and ChemGene HLD_4_L did not.

**Supplementary Information:**

The online version contains supplementary material available at 10.1007/s00414-024-03232-0.

## Introduction

PCR has been an essential and integrated part of forensic genetic investigations since the 1990s [1]. It allows identification of genetic information from small amounts of material and increases the sensitivity of DNA investigations. PCR generates millions of copies of the target loci, which makes it easier to detect the genotypes, but the many DNA copies also increase the risk of amplification of DNA from contaminated laboratory spaces and instruments. Therefore, measures to prevent contamination from PCR products were introduced and quickly became the standard in PCR laboratories. These measures include psychical separation of pre- and post-PCR areas and instrumentation, unidirectional workflows, increase air pressure in pre-PCR laboratories, reduce air pressure in post-PCR laboratories, protective clothing (gloves, hair net, shoe covers, laboratory coats), one-time use of disposable plasticware, duplicate typing, and regular test for contamination in laboratory areas [2–5]. One important aspect of reducing the risk of contamination is frequent cleaning of laboratory spaces and equipment. Common cleaning agents, sterilization methods, or ultraviolet light are typically used for decontamination. However, their effects may vary considerably [6–11].

A recent discussion on the use of cleaning reagents in our laboratory inspired us to make a survey of cleaning protocols in ten other forensic genetic laboratories. The result from this survey is shown in Supplementary Tables 1 and 2. The survey revealed that all ten laboratories used different protocols and thus, we decided to test the various methods to identify the most efficient protocol(s) for decontamination of DNA from laboratory surfaces.

## Materials and methods

A survey on cleaning protocols in forensic genetic laboratories was conducted by contacting laboratories by e-mail and by hand-outs at the 29th congress of the International Society of Forensic Genetics (2022). The survey included a questionnaire on the cleaning protocols in pre-PCR and post-PCR laboratories, how frequent specific areas (floor, contact points, LAF bench, fume hood, cabinets, instruments) were cleaned and with which reagent(s). We received answers from ten European laboratories (originating from The Netherlands, Estonia, Finland, United Kingdom, Scotland, Slovenia, Sweden, Norway, and Denmark). Their replies are shown in Supplementary Tables 1 and 2.

### Test of cleaning protocols

AmpliSeq™ libraries (Thermo Fisher Scientific) from a study of microhaplotypes (publication in preparation) were constructed according to the manufacturer’s protocol. The samples were selected from the ‘Section of Forensic Genetics anonymous collection of samples’ (RAASP-D) (j. no. 004–0065/21-7000). All samples were fully anonymised. The study follows the policy from the National Science Ethics Committee in Denmark (https://en.nationaltcenterforetik.dk) and complies with the rules of the General Data Protection Regulation (Regulation (EU) 2016/679). The libraries were quantified using the Qubit 3.0 (Thermo Fisher Scientific) and 10 µL 0.5 ng/µL library or 10 µL water (negative control) were pipetted on to a hard and clean surface in a room that had never been used for laboratory work. Squares of 2 cm^2^ were cut out in paper to mark the positions. The droplets were left to dry for 45 minutes. The surfaces in the squares were cleaned by 1) administrating the liquid cleaning reagent in Tables 1 to an absorbent Sitrix V1 wipe (ImteX Aps) and rubbing the surface, 2) rubbing the surface with the isopropanol wipes (Advanced Technology Cleaning), or 3) the surface was not cleaned (positive control). The surfaces were left until dried (app. 30 min). After cleaning, one Puritan Sterile Cotton Tip Applicator (Puritan) with 20 µL molecular grade water was used to swab the surface in each square. Subsequently, the cotton swabs were extracted using the QIAamp® DNA Blood Mini Kit (Qiagen) and the DNA Purification protocol for Buccal swabs (Spin Protocol). Finally, the extracts were quantified by real-time PCR using the QIAseq™ Library Quant Assay Kit for quantification of Ion Torrent™ libraries (Qiagen). All cleaning protocols were tested in triplicates. All qPCRs were performed in duplicates and with two different dilutions (2,000 and 20,000) yielding a total of four quantification results per sample.

**Table 1:** The percentages of recovered DNA after cleaning

Treatment	Active reagent	DNA recovered* (%)
Positive control	-	100 ± 10.3
Negative control	-	0
0.1% bleach	Hypochlorite (NaClO)	1.36 ± 0.3
0.3% bleach	Hypochlorite (NaClO)	0.66 ± 0.2
1% bleach	Hypochlorite (NaClO)	0
3% bleach	Hypochlorite (NaClO)	0
10% bleach	Hypochlorite (NaClO)	0
70% ethanol	Ethanol	4.29 ± 1.2
Water and 70% ethanol^‡^	Ethanol	0.2 ± 0
Isopropanol wipe	Isopropanol	9.23 ± 0.5
Liquid isopropanol	Isopropanol	87.99 ± 7.4
1% Virkon®	Oxidation (KHSO_5_)	0
DNA AWAY™	Alkaline (NaOH)	0.03 ± 0
5% ChemGene HLD_4_L^†^	Oxidation	1.82 ± 0.4
2 × 5% ChemGene HLD_4_L^†^ and isopropanol wipe^‡^	Oxidation/isopropanol	0.17 ± 0

* 100x(mean amount of extracted DNA/mean amount of extracted DNA from the positive control)

^†^ The product contains a combination of alcohols, amines, ammonium compounds, and chlorhexidine

^‡^ The surface was wiped twice, once with each reagent

## Results and discussion

Ten European laboratories participated in the survey and replied to questions on their cleaning protocols in pre-PCR and post-PCR laboratories (Supplementary Tables 1 and 2, respectively). In the pre-PCR laboratories, contact points, workspaces, and instruments were typically cleaned once every day, whereas the floor was cleaned once every week, and cabinets once or twice every year. In post-PCR laboratories, the frequencies of cleaning were similar, although some laboratories cleaned their instruments and contact point less frequently than in their pre-PCR area(s). In contrast, there were absolutely no consensus on the cleaning reagents. Most laboratories used their choice of reagents on all contact points, workspaces, and instruments, although there were minor differences between pre- and post-PCR areas, but none of the laboratories used the same cleaning reagent(s).

We decided to test the various cleaning protocols (in triplicate) by contaminating clean surfaces with 5 ng massively parallel sequencing (MPS) DNA libraries. The samples were left to dry, and the area was subsequently cleaned with wipes containing one of the reagents shown in Table 1. A cotton swab was used to collect the DNA from the area and the DNA was quantified in quadruplicate using the QIAseq™ Library Quant Assay Kit.

The results (Table 1) showed that all amplifiable DNA was removed by freshly made household bleach in concentrations down to 1%. At lower concentrations of bleach, some DNA was recoverable. The bleach contained 30–60% hypochlorite according to the chemical datasheet, thus, 0.3–0.6% hypochlorite was sufficient to decontaminate the surfaces. Also, the disinfectant Virkon® removed all traces of amplifiable DNA, whereas the sodium hydroxide in DNA AWAY™ left small traces of DNA. Other disinfectants, including ethanol, isopropanol, and ChemGene HLD_4_L were not successful in removing all the DNA, although they reduced the amount of DNA that was recovered. Cleaning the area multiple times with different reagents (e.g. water and ethanol) reduced the amount of recovered DNA further, however, some DNA were still present in these areas after the area was wiped off two or three times.

The results shown here support the conclusions from previous studies [6, 8–11] that hypochlorite and Virkon® are efficient cleaning agents. However, hypochlorite may produce poisonous chlorine gases if it reacts with acidic solutions and key components in several commercial extraction kit [8]. Furthermore, hypochlorite is corrosive against metals, and cleaning the surface with 70% EtOH or water after cleaning with hypochlorite has been recommended [6, 8, 11]. However, none of the laboratories in our study used these cleaning protocols and they were not tested here. Virkon® is a strong oxidative agent and may also generate halogen gasses if it is in contact with halide compounds. It is less corrosive than hypochlorite and had the best decontamination efficiency on blood deposits on plastic, metal, and wood [11]. Standard protective equipment (protective gloves, laboratory coats, safety glasses) are recommended for both hypochlorite and Virkon® according to their material safety data sheets, and the cost of the two products are small, although household bleach will be cheaper than Virkon® when it is used in diluted form. Virkon® is less toxic for the environment than bleach, which may be an important detail when choosing between the two products.

Disinfection of pre-PCR areas after handling of samples from crime scenes is sensible and may protect the laboratory personnel from infections. However, not all the tested disinfectants in this study removed the DNA. As shotgun sequencing becomes more widespread in forensic genetics, the DNA from all organisms, and not only human DNA, may contaminate a trace sample and the interpretation of the sequencing results. Therefore, cleaning with a disinfectant, that do not remove the DNA, should be followed by cleaning with a reagent that does.

## Conclusions

Removal of DNA from all surfaces in forensic genetic laboratories is crucial to avoid cross-sample contamination. Freshly made household bleach and Virkon® appeared to be the most efficient reagents for decontamination of laboratory surfaces, whereas DNA AWAY™ and the disinfectants ethanol, isopropanol, and ChemGene HLD_4_L only removed some of the DNA.

### Electronic supplementary material

Below is the link to the electronic supplementary material.

Supplementary Material 1

Supplementary Material 2
