# Supplementary material for: Cleaning protocols in forensic genetic laboratories
Source: Int J Legal Med. 2024 Apr 23;138(5):1787–90. doi: 10.1007/s00414-024-03232-0 (PMC11306349; doi:10.1007/s00414-024-03232-0)
Supplement: Supplementary file 1 — Supplementary Material 1 [file 414_2024_3232_MOESM1_ESM.pdf]

Supplementary Table 1. Responses from questionaire on cleaning of prePCR areas.

		Floor		Contact points		LAF bench		Fume hood		Cabinets		Instruments	
Laboratory number	Accreditation	Frequency	Reagents	Frequency	Reagents	Frequency	Reagents	Frequency	Reagents	Frequency	Reagents	Frequency	Reagents
1	ISO17025	1 time / day	Detergent	1 time / day	3 % bleach solution	-	-	1 time / day	3 % bleach solution	1 time / year	-	1 time / day	3 % bleach solution
2	ISO17025	1 time / week	Detergent	1 time / week	0.3% sodium hypochlorite	1 time / week	0.3% sodium hypochlorite	1 time / week	0.3% sodium hypochlorite	1 time / week	0.3% sodium hypochlorite	Before and after use	0.3% sodium hypochlorite + UV light
3	-	2 times / week	Water	1 time / day	70 % ethanol + UV light	1 time / day	70 % ethanol + UV light	1 time / day	70 % ethanol + UV light	2 times / year	Detergent	1 time / day	70 % ethanol
4	ISO17025	1 time / day	Detergent	1 time / day	ChemGene HLD4L 5%[†] or DNA AWAY™[‡]	1 time / day	ChemGene HLD4L 5%[†]	1 time / day	ChemGene HLD4L 5%[†]	2 times / year	ChemGene HLD4L 5%[†]	1 time / day	Water + 70 % ethanol
5	ISO17025	1 time / week	10 % bleach solution	1 time / week	10 % bleach solution	1 time / day	70 % ethanol	1 time / day	70 % ethanol	2 times / year	10 % bleach solution	1 time / day	10 % bleach solution
6	Accreditation in progress / ISO/IEC 17025:2017	1 time / week	Detergent	1 time / week	2x ChemGene spray[†] followed by Azowipes 70% isopropanol wipe.	-	-	-	-	1 time / week	2x ChemGene[†] spray followed by 1x wipe of ChemGene disinfectant wipe, alcohol free.	1 time / day	2x ChemGene spray[†] followed by Azowipes 70% isopropanol wipe.
7	ISO17025	1 time / week	Detergent	1 time / day	0.1 % bleach solution	1 time / day	70 % ethanol or 0.1 % beach solution + UV light	1 time / day	0.1 % bleach solution	-	-	1 time / day	70 % ethanol or 0.1 % beach solution
8	ISO17025	1 time / week	Detergent	1 time / day	Virkon 1 %[§]	1 time / day	Virkon 1 %[§]	-	-	1 time / week	Virkon 1 %[§]	1 time / day	Virkon (1%)[§] or Microsol (10%)[$] for metal/moving parts
9	ISO17025	3 times / week	Detergent	-	-	1 time / day	Incides^ or Spitaderm[£] or 70 % ethanol	1 time / day	Incides^ or Spitaderm[£] or 70 % ethanol	1 time / day	Incides^ or Spitaderm[£]	1 time / day	Incides^ or Spitaderm[£] and UV or sometimes 4 % chlorine solution or 70% ethanol
10	ISO17025	1 time / day	Detergent	1 time / week	Detergent or DAX Disinfectant*	-	-	1 time / day	DAX Disinfectant*	-	-	1 time / day	DAX Disinfectant*

† ChemGene contains a combination of alcohols, amines, ammonium compounds, and chlorhexidine
