# Supplementary material for: Cleaning protocols in forensic genetic laboratories
Source: Int J Legal Med. 2024 Apr 23;138(5):1787–90. doi: 10.1007/s00414-024-03232-0 (PMC11306349; doi:10.1007/s00414-024-03232-0)
Supplement: Supplementary file 2 — Supplementary Material 2 [file 414_2024_3232_MOESM2_ESM.pdf]

Supplementary Table 2. Responses from questionaire on cleaning of postPCR areas.

		Floor		Contact points		LAF bench		Fume hood		Cabinets		Instruments	
Laboratory number	Accreditation	Frequency	Reagents	Frequency	Reagents	Frequency	Reagents	Frequency	Reagents	Frequency	Reagents	Frequency	Reagents
1	ISO17025	1 time / week	Detergent	1 time / day	3 % bleach solution	1 time / week	3 % bleach solution	1 time / day	80 % ethanol	1 time / year	3 % bleach solution	1 time / week	3 % bleach solution or 80 % ethanol
2	ISO17025	1 time / week	0.3% sodium hypochlorite	1 time / week	0.3% sodium hypochlorite	1 time / week	0.3% sodium hypochlorite	1 time / week	0.3% sodium hypochlorite	1 time / week	0.3% sodium hypochlorite	Before and after use	0.3% sodium hypochlorite
3	-	1 time / week	Water	1 time / day	70 % ethanol	-	-	2 times / year	Detergent or 70 % ethanol or isopropanol wipes	2 times / year	Detergent or 70 % ethanol or isopropanol wipes	1 time / day	70 % ethanol
4	ISO17025	1 time / day	Detergent	1 time / week	70 % ethanol	-	-	1 time / day	70 % ethanol & milli-Q-water or ChemGene HLD4L 5%[†]	2 times / year	ChemGene HLD4L 5%[†]	1 time / week	Vacuuming, 70 % ethanol & milli-Q-water
5	ISO17025	1 time / week	10 % bleach solution	1 time / week	10 % bleach solution	-	-	1 time / day	70 % ethanol	2 times / year	10 % bleach solution	1 time / week	10 % bleach solution
6	Accreditation in progress / ISO/IEC 17025:2017	-	-	1 time / month	2x ChemGene spray[†] followed by Azowipes 70% isopropanol wipe.	-	-	-	-	-	-	1 time / month	2x ChemGene spray[†] followed by Azowipes 70% isopropanol wipe.
7	ISO17025	1 time / week	Detergent	1 time / day	0.1 % bleach solution	1 time / day	70 % ethanol or 0.1 % beach solution + UV light	-	-	-	-	1 time / day	70 % ethanol or 0.1 % beach solution
8	ISO17025	1 time / week	Detergent	1 time / week	Virkon 1 %[§]	-	-	-	-	1 time / week	Virkon 1 %[§]	1 time / week	Virkon (1%)[§] or Microsol (10%)[$] for metal/moving parts
9	ISO17025	3 times / week	Detergent	-	-	1 time / day	Incides^ or Spitaderm[£] or 70 % ethanol	1 time / day	Incides^ or Spitaderm[£] or 70 % ethanol	1 time / day	Incides^ or Spitaderm[£]	1 time / day	Incides^ or Spitaderm[£] and UV or sometimes 4 % chlorine solution or 70% ethanol
10	ISO17025	1 time / day	Detergent	1 time / week	Detergent or DAX Disinfectant*	-	-	-	-	-	-	1 time / week	DAX Disinfectant*

† ChemGene contains a combination of alcohols, amines, ammonium compounds, and chlorhexidine
